# Injuries in Australian Army full-time and part-time personnel undertaking basic training

**DOI:** 10.1186/s12891-018-2390-2

**Published:** 2019-01-05

**Authors:** Ben Schram, Rodney Pope, Robin Orr

**Affiliations:** 1 Tactical Research Unit, Bond University, Robina, Australia; 2 School of Community Health, Charles Sturt University, Albury, Australia

**Keywords:** Recruit, Military, Physical training, Knee, Muscle stressing

## Abstract

**Background:**

Musculoskeletal injuries are a problem in military personnel as they detract from force readiness and may prevent deployment. Injuries occur during basic training at three times the rate observed in post-training military service and more commonly in part time (PT) when compared to full time (FT) army personnel. The purpose of this study was to examine differences in rates and patterns of reported injuries between full time (FT) and part time (PT) personnel undertaking army basic training.

**Methods:**

A retrospective cohort study was conducted to determine and compare rates and patterns of injuries which occurred during basic training in PT and FT personnel. Injury data from the period 01 July 2012 to 30 June 2014 was obtained in a non-identifiable format from the Workplace Health, Safety, Compensation and Reporting (WHSCAR) database of the Australian Department of Defence. Analysis included descriptive statistics and the calculation of injury rates and injury rate ratios.

**Results:**

A total of 1385 injuries were reported across FT and PT cohorts, with an injury rate ratio for FT:PT of 1.06 [0.80–1.40], when accounting for exposure. In FT personnel, 1192 (90%) were Minor Personal Injuries (MPIs) and 43 (3.2%) Serious Personal Injuries (SPIs). In PT personnel, 147 (94.8%) were MPIs and three (1.9%) SPIs. In both FT and PT personnel, injuries most commonly: occurred during Physical Training (41.7% FT, 515 MPIs, 10 SPIs, 32% PT. 48 MPIs, 1 SPI); affected the knee (FT 41.7% 159 MPIs, 7 SPIs, PT 36.0%, 22 MPIs, 0 SPIs); involved soft tissue damage (FT 60.9%, 744 MPIs, 8 SPIs, PT 69.3%, 103 MPIs, 1 SPI); and were due to muscular stress (FT 41.7%, 509 MPIs, 6 SPIs, PT 36%, 54 MPIs, 0 SPIs).

**Conclusions:**

FT and PT recruits exhibited similar injury profiles, with mechanisms, sites and types of injuries in agreement with other research. Given these similarities, effective interventions that reduce injury risks in either population will likely benefit both.

## Background

Musculoskeletal injuries are a major problem in military personnel as they detract from force readiness, may prevent soldiers from deploying and create a high financial burden [1, 2]. Injury rates during military basic training have been estimated to range between 6 and 30 per 100 personnel per month in full-time personnel, contributing to attrition and increased training costs [3–5]. These injury rates in basic training are around three times those experienced at other times during a military career [4, 6]. Therefore, prevention of injuries during military training is a priority, with injuries occurring during basic training of particular interest [7].

Basic training is a particularly important phase of training in which to prevent injuries, as any injury occurring in this phase will become a risk factor for future injury [2]. Injury risk among recruits is known to be related to poor upper body endurance, low aerobic fitness [8] and prior injury history [9]. A recruit’s exercise load during basic training is often much greater than that which they experienced during prior civilian life, putting them at risk of overuse and other injuries during their initial training period [10]. Low initial levels of fitness will exacerbate this risk [4], and low levels of fitness in army recruits upon enlistment may be reflective of the general population, with the endurance performance of male recruits decreasing over the years in many countries [2, 11]. Previous research in army recruits has shown that the lower limbs are the most common locations of musculoskeletal injuries that occur during basic training [12, 13] and that overuse injuries, thought to be caused by the sudden increase in intense physical activity such as running, marching and drill, are the most common type [14, 15].

A range of interventions have been trialled to reduce injury rates in basic training, including for example fitness screening [2] and pre-conditioning programs [16]. However, so that interventions can be implemented in a targeted fashion, a thorough understanding of which activities and locations are associated with higher risk is required [3]. With this in mind, one particular issue that has not yet been researched is differences in injury rates and patterns between full time and part-time (reserve) personnel undertaking basic training programs. Any such differences need to be ascertained in order to guide interventions in each of these populations, and particularly in part-time personnel, who to date have been under-researched with regard to injuries and their prevention. In the Australian Army, basic training for part-time personnel is, at the time of writing, four weeks long, whereas basic training for full time personnel is 12 weeks in duration. Some discrepancies exist between part-time and full-time personnel in the fitness improvements they achieve in basic training [17]. Recent investigations at an army-wide level have also found that injury rates may be higher per unit time served for part-time personnel than for full-time personnel [5]. This difference is thought to possibly be due to less chronic conditioning of reserve personnel, in the longer term [18].

Given that basic training constitutes a high risk period for injury amongst army personnel, that there is currently a difference in training duration between full-time and part-time personnel, and that injury rates appear higher overall for part-time army personnel, the aim of this investigation was to investigate and compare the rates and patterns of injuries suffered by both full-time Australian Regular Army (ARA) and part-time Australian Army Reserve (ARES) personnel during basic training.

## Methods

A retrospective cohort study was conducted to determine and compare the patterns and rates of injuries which occurred during basic training in both ARA and ARES personnel. Injury data from the period 01 July 2012 to 30 June 2014 was obtained in a non-identifiable format from the Workplace Health, Safety, Compensation and Reporting (WHSCAR) database of the Australian Department of Defence. This database constitutes the record of all occupational health and safety incidents, including those leading to injuries, experienced by army personnel [19]. This database contained information on service type, type of occurrence, date of incident, nature of resulting injury, body site affected by resulting injury, mechanism of resulting injury, activity at the time of the incident (including specific event, e.g. field exercise, if applicable), duty status and an incident description.

Injury records were included in the study if they related to army basic training and if they represented a minor or serious personal injury, as per the Department of Defence definitions [19]. It was planned that injury records would be excluded if they contained missing or incomplete data, however no records were missing data and so none were excluded on this basis. Outcomes of interest included nature of injury, injury mechanisms, activity being performed at the time of injury and body location of injury.

The Australian Defence Human Research Ethics Committee (ADHREC, LERP14–024) and the Bond University Human Research Ethics Committee (BUHREC, RO-1907) granted ethics approval for this study. Departmental authorisation for the project was obtained in parallel to the process for obtaining ADHREC approval. Authorisation to publish this study was obtained from the Australian Defence Force Joint Health Command.

### Data analysis

The WHSCAR data were manually cleaned, following receipt, to ensure that only eligible injury records were retained, with each line of data reviewed and duplicates removed. Each data record was further verified, corrected or made more precise by manually comparing the allocated Type of Occurrence Classification System (TOOCS) classifications with the free text narrative data from the same record [20]. When discrepancies were identified, precedence was given to the free text narratives and the TOOCS classification was adjusted accordingly, as narratives provided by incident reporters are considered more detailed and accurate than data entered by a third party using a finite coding system [20]. The adjusted dataset was employed in the data analysis. To increase data accuracy, brevity and sensitivity, some TOOCS fields were aggregated, notably ‘nature of injury’ and ‘activity’. In the TOOCS nature of injury codes, ‘soft tissue injuries due to trauma or unknown mechanism’ subsumed ‘trauma to muscle’ [a soft tissue] and ‘trauma to tendons’ [another soft tissue]. In addition, ‘Trauma to joints and ligaments’ incorporated ‘Trauma to joints and ligaments not elsewhere classified’ and ‘Trauma to joints and ligaments unspecified’. In the TOOCS ‘activity’ codes, all sports were merged into an aggregated classification, ‘sport’. ‘Running´ was subsumed by ‘physical training’, and ‘patrolling’ and all weapon handling activities were subsumed by ‘combat training’.

Descriptive analyses, including analysis of frequencies and proportions, were then undertaken using SPSS [21] to establish and enable visual and narrative comparisons of the numbers and patterns of injuries reported by full-time and part-time personnel to have occurred during army basic training.

Injury rates (IRs) were then calculated per 100 recruits who completed a single training period (80 days for ARA and 28 days for ARES) and per 100 full-time-equivalent (FTE) years of training, the latter to allow for valid comparisons of injury rates between the ARA and ARES cohorts by accounting for the different ARA and ARES basic training program durations. For this purpose, one FTE year of training was assumed to equate to 232 days (365 days in a year – 104 days of weekends – 20 days of annual leave – 9 days of public holidays). Injury rate ratios (IRRs) for ARA:ARES were subsequently calculated by dividing the injury rates (injuries per 100 full-time-equivalent [FTE] years of training) for the ARA cohort by those for the ARES cohort [22]. The 95% confidence intervals around the IRRs were also calculated, as follows [22]:

$$ 95\%\mathrm{CI}=\exp\ \left(\ln \left[\mathrm{IRR}\right]-1.96\mathrm{xSE}\left(\ln \left[\mathrm{IRR}\right]\right)\right)\ \mathrm{to}\ \exp\ \left(\ln \left[\mathrm{IRR}\right]+1.96\mathrm{xSE}\left(\ln \left[\mathrm{IRR}\right]\right)\right) $$

where

$$ \mathrm{SE}\left(\ln \left[\mathrm{IRR}\right]\right)=\surd \left(1/\left[\mathrm{IRARA}\right]+1/\left[\mathrm{IRARES}\right]-1/\mathrm{nARES}-1/\mathrm{nARA}\right) $$

## Results

In total, 1385 injuries were reported during basic training across the ARA and ARES cohorts combined. The numbers of recruits, estimated FTE years of training and injury rates for the ARA and ARES cohorts are provided in Table 1, below. Of these injuries, 1235 (89%) affected ARA recruits and 150 (11%) affected ARES recruits. Despite a higher injury rate in ARA recruits than in ARES recruits when the injury rates were expressed per 100 recruits who completed the respective periods of training (ARA 34.5: ARES 9.2), once the injury rates were transformed to be expressed per 100 FTE years of training, thereby accounting for training exposure, they were much more similar (ARA 80.5: ARES 76.1), resulting in an overall IRR of 1.06 [95% CI 0.80–1.40].

**Table 1:** ARES and ARA personnel, estimated person years of service, injury numbers and injury rates (per 100 recruits [per 100 FTE years of training])

	ARA	ARES	Combined
Personnel (2012–2014)	4452 (73.2%)	1630 (26.8%)	6082 (100%)
FTE years of service	1535 (88.6%)	197 (11.4%)	1732 (100%)
Total Injuries	1235 (89%)	150 (11%)	1385 (100%)
Injury Rates	27.7 [80.5]	9.2 [76.1]	22.8 [80.0]

*ARA* Australian Regular Army, *ARES* Army Reserve, *FTE* Full Time Equivalent

Of the ARA injuries, 1192 (97%) were Minor Personal Injuries (MPIs) and 43 (3%) were Serious Personal Injuries (SPIs). Amongst ARES recruits, 98% of injuries were MPI and 2% were SPI (Table 2). When adjusted for training exposure, ARA personnel suffered slightly more MPIs (IRR = 1.04) and almost twice as many SPIs as ARES personnel (IRR = 1.87).

**Table 2:** Summary of injuries reported by ARA and ARES recruits to have occurred during basic training in the Australian Army, in the period 1 July 2012 to 30 June 2014 (n, IR calculated as injuries per 100 recruits, [IR calculated as injuries per 100 FTE years of training])

Incident Type	ARA		ARES		Combined		ARA:ARES
	n	IR	n	IR	n	IR	IRR [95%CI]
Minor Personal Injury	1192	26.77 [77.7]	147	9.02 [74.6]	1339	11.01 [77.3]	1.04 [0.79–1.38]
Serious Personal Injury	43	0.97 [2.8]	3	0.18 [1.5]	46	0.38 [2.7]	1.87 [0.26–13.5]
Total	1235		150		1385		

*ARA* Australian Regular Army, *ARES* Army Reserve

*IR* injury rate, *IRR* injury rate ratio

The activities which caused injuries during basic training are listed in Table 3. The most common activity in which injuries occurred was physical training, accounting for 43.2% of all MPIs and 23.3% of all SPIs in the ARA recruits and 32.6% of all MPIs and 33.3% of all SPIs in the ARES recruits. Combat training was the second most common activity to give rise to injuries, with 22.2% of MPIs and 16.3% of SPIs in the ARA recruits and 27.9% of MPIs and no SPIs in the ARES recruits arising from this activity type. Marching was the third most common identified activity followed by unknown activities. Unknown activities were frequently reported when an injury had an insidious onset, in which a specific activity causing the injury could not be determined. Such injuries included, for example, ingrown toenails, blisters and injuries which were listed as occurring during ‘recruit training’ rather than during a specific activity. Of the 14 SPIs for which the activity was listed as ‘unknown’, 11 involved appendicitis, pneumonia or tonsillitis, with the remaining three being musculoskeletal injuries without an attributable activity (i.e. an overuse injury).

**Table 3:** Activities reported by ARA and ARES recruits to have been undertaken at the time injuries occurred during basic training in the Australian Army, in the period 1 July 2012 to 30 June 2014

	ARA		ARES		IRR (ARA/ARES) [95% CI]
Activity	MPI	SPI	MPI	SPI	
Physical Training (PT)	515 (40.6%)	10 (0.8%)	48 (32.0%)	1 (0.7%)	1.37 [0.84–2.25]
Combat Training	265 (20.0%)	7 (0.6%)	41 (27.3%)	0 (0.0%)	0.85 [0.46–1.57]
Marching	117 (9.5%)	3 (0.2%)	16 (10.7%)	0 (0.0%)	0.96 [0.36–2.54]
Unknown	117 (9.5%)	14 (1.1%)	17 (11.3%)	2 (1.3%)	0.88 [0.36–2.19]
Walking	54 (4.4%)	3 (0.2%)	6 (4.0%)	0 (0.0%)	1.22 [0.27–5.50]
Manual/Materials Handling	27 (2.2%)	1 (0.1%)	5 (3.3%)	0 (0.0%)	0.72 [0.11–4.78]
Sitting/standing	14 (1.1%)	1 (0.1%)	0 (0.0%)	0 (0.0%)	–
Swimming	14 (1.1%)	1 (0.1%)	4 (2.7%)	0 (0.0%)	0.48 [0.04–5.34]
Construction/Engineering	12 (1.0%)	0 (0.0%)	1 (0.7%)	0 (0.0%)	1.54 [0.05–52.39]
Personal Hygiene	10 (0.8%)	0 (0.0%)	0 (0.0%)	0 (0.0%)	–
Weapons familiarisation	9 (0.7%)	0 (0.0%)	5 (3.3%)	0 (0.0%)	0.23 [0.01–3.93]
Sensitive	8 (0.6%)	0 (0.0%)	0 (0.0%)	0 (0.0%)	–
Cleaning	5 (0.4%)	0 (0.0%)	2 (1.3%)	0 (0.0%)	0.32 [0.01–16.53]
Patient/Patient Care	5 (0.4%)	0 (0.0%)	0 (0.0%)	0 (0.0%)	–
Collated Others*	20 (1.6%)	3 (0.2%)	2 (1.3%)	0 (0.0%)	1.47 [0.12–18.21]
Total	1192 (96.5%)	43 (3.5%)	147 (98.0%)	3 (2.0%)	

*Collated others: Collation of remaining activities with less than three (*n* = 3) occurrences. *ARA* Australian Regular Army, *ARES* Army Reserve, *MPI* Minor Personal Injury, *SPI* Serious Personal Injury, *IRR* Injury Rate Ratio

Expressed as raw number (% of all injuries for that service type)

The most common body location of injuries reported by recruits as having occurred during basic training was the knee, in both ARA recruits (13.3% of MPIs and 16.3% of SPIs) and ARES recruits (14.9% of MPIs and 0 reported SPIs; Table 4). The ankle and lower leg together comprised the next two most commonly injured body locations in ARA and ARES recruits. Despite no reported SPIs in either ARA or ARES recruits for the ankle or lower leg, the ankle and lower leg accounted for 11.5 and 10.3%, respectively, of ARA recruit MPIs and for 8.2 and 11.6%, respectively, of ARES recruit MPIs. Of the 21 SPIs listed as ‘collated others’, 10 were systemic illnesses, six were to the head due to concussions, and several single entries represented injury to the eye, pelvis, neck, abdomen and what was listed as ‘multiple locations’.

**Table 4:** Body locations of injuries reported by ARA and ARES recruits to have occurred during basic training in the Australian Army, in the period 1 July 2012 to 30 June 2014. Expressed as raw number (% of all injuries for that service type)

	ARA		ARES		IRR (ARA/ARES) [95% CI]
Location	MPI	SPI	MPI	SPI	
Knee	159 (12.9%)	7 (0.6%)	22 (14.7%)	0 (0.0%)	0.97 [0.42–2.20]
Ankle	137 (11.1%)	0 (0.0%)	12 (8.0%)	0 (0.0%)	1.46 [0.53–4.05]
Lower Leg	123 (10.0%)	0 (0.0%)	17 (11.3%)	0 (0.0%)	0.93 [0.36–2.40]
Foot	121 (9.8%)	4 (0.3%)	7 (4.7%)	0 (0.0%)	2.29 [0.66–7.88]
Shoulder	84 (6.8%)	3 (0.2%)	11 (7.3%)	1 (0.7%)	0.93 [0.3–2.89]
Lower Back	64 (5.2%)	0 (0.0%)	10 (6.7%)	0 (0.0%)	0.82 [0.23–2.97]
Upper Leg	37 (3.0%)	0 (0.0%)	12 (8.0%)	0 (0.0%)	0.40 [0.09–1.74]
Hip	36 (2.9%)	0 (0.0%)	2 (1.3%)	0 (0.0%)	2.31 [0.23–23.53]
Abdominal Muscles	34 (2.8%)	2 (0.2%)	2 (1.3%)	1 (0.7%)	1.54 [0.20–11.75]
Lower Limb – Multiple	32 (2.6%)	1 (0.1%)	0 (0.0%)	0 (0.0%)	–
Back – unspecified	27 (2.2%)	0 (0.0%)	5 (3.3%)	0 (0.0%)	0.69 [1.10–4.70]
Lower Limb – unspecified	27 (2.2%)	2 (0.2%)	6 (4.0%)	0 (0.0%)	0.62 [0.10–3.78]
Wrist	26 (2.1%)	0 (0.0%)	3 (2.0%)	0 (0.0%)	1.11 [0.13–9.85]
Fingers	25 (2.0%)	1 (0.1%)	4 (2.7%)	0 (0.0%)	0.83 [0.11–6.37]
Circulatory System	21 (1.7%)	2 (0.2%)	3 (2.0%)	0 (0.0%)	0.98 [0.10–9.32]
Collated Others*	239 (19.4%)	21 1.7%)	31 (20.7%)	1 (0.7%)	1.04 [0.54–2.02]
Total	1192 (96.5%)	43 (3.5%)	147 (98.0%)	3 (2.0%)	

*Collated Others: Collation of remaining locations with less than 20 (*n* = 20) occurrences. *ARA* Australian Regular Army, *ARES* Army Reserve, *MPI* Minor Personal Injury, *SPI* Serious Personal Injury, *IRR* Injury Rate Ratio

The most common natures of injuries are presented in Table 5. Soft tissue injuries were by far the most common nature of injury for both ARA and ARES recruits, accounting for 61.8% of all reported injuries. They accounted for 62.4% of ARA recruit MPIs, 18.6% of ARA recruit SPIs, 70.1% of ARES recruit MPIs and 33.3% of ARES recruit SPIs.

**Table 5:** Natures of injuries reported by ARA and ARES recruits to have occurred during basic training in the Australian Army, in the period 1 July 2012 to 30 June 2014. Expressed as raw number (% of all injuries for that service type)

	ARA		ARES		IRR (ARA/ARES) [95% CI]
	MPI	SPI	MPI	SPI	
Soft tissue injuries	744 (60.2%)	8 (0.5%)	103 (68.7%)	1 (0.7%)	0.93 [0.65–1.33]
Other fractures	101 (8.2%)	4 (0.3%)	2 (1.3%)	0 (0.0%)	6.73 [0.84–53.75]
Laceration or open wound	46 (3.7%)	2 (0.2%)	8 (5.3%)	0 (0.0%)	0.77 [0.18–3.33]
Trauma to joints and ligaments	34 (2.8%)	2 (0.2%)	3 (2.05)	0 (0.0%)	1.54 [0.20–11.75]
Contusion, bruising and superficial crushing	32 (2.6%)	0 (0.0%)	6 (4.0%)	0 (0.0%)	0.68 [0.12–3.95]
Dislocation	28 (2.3%)	4 (0.3%)	2 (1.3%)	1 (0.7%)	1.37 [0.17–10.98]
Friction Burn	24 (1.9%)	0 (0.0%)	5 (3.3%)	0 (0.0%)	0.62 [0.08–4.48]
Other soft tissue diseases	23 (1.9%)	0 (0.0%)	0 (0.0%)	0 (0.0%)	–
Collated Others*	160 (13.0%)	23 (1.9%)	18 (12.0%)	1 (0.7%)	1.23 [0.54–2.85]
Total	1192 (96.5%)	43 (3.5%)	147 (98.0%)	3 (2.0%)	

*Collated Others: Collation of other natures of injury with less than 20 (n = 20) occurrences. *ARA* Australian Regular Army, *ARES* Army Reserve, *MPI* Minor Personal Injury, *SPI* Serious Personal Injury, *IRR* Injury Rate Ratio

Muscular stress with no objects being handled, falls and muscular stress from lifting, carrying or putting objects away were the most prevalent mechanisms of injury reported by both ARA and ARA recruits (Table 6). These three mechanisms of injury accounted for 72.6% of all injuries suffered during basic training. Muscular stress with no objects being handled accounted for 42.7% of ARA recruit MPIs, 13.9% of ARA recruit SPIs, 36.7% of ARES recruit MPIs and no reported ARES recruit SPIs.

**Table 6:** Mechanisms of injuries reported by ARA and ARES recruits to have occurred during basic training in the Australian Army, in the period 1 July 2012 to 30 June 2014. Expressed as raw number (% of all injuries for that service type)

	ARA		ARES		IRR (ARA/ARES) [95% CI]
	MPI	SPI	MPI	SPI	
Muscular stress with no objects being handled	509 (41.2%)	6 (0.5%)	54 (36.0%)	0 (0.0%)	1.22 [0.75–1.98]
Falls	192 (15.5%)	5 (0.4%)	29 (19.3%)	1 (0.7%)	0.84 [0.41–1.74]
Muscular stress while lifting, carrying, or putting objects away	182 (14.7%)	8 (0.6%)	20 (13.3%)	0 (0.0%)	1.22 [0.54–2.75]
Other and multiple mechanisms of incident	67 (5.4%)	12 (1.0%)	13 (8.7%)	1 (0.7%)	0.72 [0.23–2.23]
Contact with moving or stationary objects	69 (5.6%)	1 (0.1%)	9 (6.0%)	0 (0.0%)	1.00 [0.27–3.62]
Unspecified	31 (2.5%)	2 (0.2%)	6 (4.0%)	1 (0.7%)	0.60 [0.11–3.26]
Rubbing and Chafing	28 (2.3%)	0 (0.0%)	6 (4.0%)	0 (0.0%)	0.60 [0.10–3.72]
Being hit by moving object	24 (1.9%)	1 (0.1%)	1 (0.7%)	0 (0.0%)	0.53 [0.14–74.43]
Collated Others*	90 (7.3%)	8 (0.6%)	9 (6.0%)	0 (0.0%)	1.40 [0.42–4.59]
Total	1192 (96.5%)	43 (3.5%)	147 (98.0%)	3 (2.0%)	

*Collated Others: Collation of other mechanisms of injury with less than 20 (n = 20) occurrences. *ARA* = Australian Regular Army, *ARES* Army Reserve, *MPI* Minor Personal Injury, *SPI* Serious Personal Injury, *IRR* Injury Rate Ratio

## Discussion

The aim of this study was to determine and compare the rates, types, source activities, body locations, natures and mechanisms of injuries reported to have occurred in full-time and part-time army recruits during basic training. In ARA and ARES recruits, injury rates were similar, with soft tissue injuries, injuries to the knee and injuries occurring during physical training most commonly reported in both cohorts. These results are in agreement with other published studies which have found that injuries during military training are most commonly at or below the knee [3, 23], that physical training is most commonly the activity in which injury occurs [24] and that injuries to soft tissues are most common and mostly overuse in nature [23]. These, and the broader findings from this study, suggest that injury patterns are very similar between full-time and part-time recruits undertaking army basic training. Given that the types and causes of injuries are similar, it is also reasonable to expect that similar approaches to injury prevention would be effective in both full-time and part-time army recruit populations.

In line with the findings of other studies, the injury rates in basic training observed in this investigation were higher than rates reported for later periods in a military career [4, 6]. Injury rates in the wider Australian Army have been reported, based on the same data source, to be 17.7 injuries per 100 person-years of active service [5] across ARA and ARES, rates which are much lower than the 80.0 injuries per 100 FTE years of recruit training found in this study. Both ARA and ARES appear to have higher overall injury rates during basic training (ARA 80.5; ARES 76.1 injuries per 100 FTE years of recruit training) than at other times during their career (ARA 16.72; ARES 30.50 injuries per 100 years of active service [5]).

The IRR found between ARA and ARES in this investigation with respect to MPIs occurring during basic training is significantly lower, at 1.04 [95% CI 0.79–1.38], than previous reports based on the same data source of 1.87 [95% CI 1.78–1.96] for MPIs for army as a whole [5]. This difference highlights that both ARES and ARA suffer minor injuries at a similar rate at this early stage in their career. The IRR between ARA and ARES in this study for SPI occurring during basic training was higher, at 1.87 [95% CI 0.26–13.5] than the IRR yielded by other reports based on the same data source for Army as a whole, being 1.24 [95% CI 0.96–1.59], but the 95% confidence interval around the IRR estimate for army basic training in this study is notably wide, indicating this difference in IRR is not significant.

The results in this study appear to be similar to what is found globally, with 28% of US army recruits suffering an injury (71% of injuries to the lower limb) during 8 weeks of basic training [12] and 28.3% of Greek Army Officer recruits suffering an injury during a seven week course, with 75.9% of those injuries being to the lower limb [25]. Likewise, 27.6% of recruits were reported to be injured in basic training in the Maltese Army on a 135 day course, with the lower limb being most commonly injured [26]. Similarly, the low back and lower extremity accounted for 71.5% of all injuries (90% of which were overuse) that led to attrition in basic training in the Israel Defence Force [27].

Minimising injuries during basic training should therefore be a focus for both ARA and ARES alike, with close attention paid in both populations to known risk factors for injury. In basic training specifically, Knapik and colleagues have found that low aerobic capacity in army recruits leads to a greater physiological stress during endurance based training tasks such as marching, obstacle courses, drill and running [23]. Addressing this problem is a dilemma for defence forces, as despite the reported benefits of physical fitness to protect against injury, increasing physical training can cause higher injury rates, requiring a balance between the need for fitness and the increases in risk of injury associated with increases in training volume [1]. However, it should be noted that research has shown that greater improvements in strength, endurance, and balance can be achieved with the same training volume when it is delivered by more experienced instructors [11]. While this benefit is known, defence forces must make an active effort to provide opportunity to its instructors to be able to become more experienced and qualified. It is thought that more experienced, tertiary qualified physical training instructors may be better able to adapt the training contents and loads to individual needs. For example, they may be able to implement high intensity intermittent training in place of some aspects of long duration endurance training, replace individual, machine based strength training with strength circuit training, and employ balance and agility training, though the latter with care to ensure overall training load does not increase, in order to prevent an increase in injury rates [13].

There may also be scope for trained instructors to monitor recruits for movement quality during fitness assessments, and if problems are identified, scrutinise more closely their performance on components of movements screens such as the Functional Movement Screen (FMS). While completing the entire FMS for all recruits may not be feasible, it may be feasible for individual components relating to the most common injury sites (for example, inline lunge for lower limb control, or general strength testing around the knee and hip) to be addressed in those recruits who are at risk of injury due to poor performance in fitness assessments; a concept proposed by Orr et al. following associations between poor FMS sub scores and sites of reported injuries in a police population [28].

Previous research has shown that part-time army recruits tend to be significantly older, heavier and possess lower relative aerobic fitness than full-time recruits [17]. In addition, the differences in training dose in army basic training for full-time and part-time personnel can lead to a significantly greater increase in aerobic fitness by the end of basic training in full-time personnel when compared to part-time personnel [17]. Although it was not measured in this study, the shorter duration of basic training for ARES recruits may result in lower aerobic fitness gains than those made by recruits undertaking the longer ARA basic training course. This would mean that part-time personnel must improve their fitness after basic training or experience a greater level of injury risk if they are to be expected to work alongside full-time forces, completing the same tasks, in later years [29]. Recent research suggests that this heightened injury risk in part-time army personnel, per unit of time served, may in fact be a reality [5]. Further research is needed to confirm this possibility and test whether interventions designed to enhance fitness of part-time personnel are effective in reducing injury risk.

Preventative strategies for basic training injuries may also include addressing the risk factors for injury which have been reported in previous studies. These include low body mass [30], greater age [7], a prior injury [9], minimal prior physical activity [23] and cigarette smoking [7]. Other potential strategies may include utilising injury minimisation strategies which have been researched and implemented in elite sport. The tracking of both internal training load indicators (Rate of Perceived Exertion (RPE) and Heart Rate) and external training load indicators (speed, distance covered, impacts etc) has assisted in decreasing injury rates in professional sport [31] and is an approach which could potentially be utilised in this population. One method of quantifying training load is multiplying RPE by session duration to give a exertional minute measure [31]. In elite sport, where both injuries and training loads have been researched further, increases in internal training load indicators of greater than 1750 units per week or 4000 units per fortnight have been shown to increase the risk of injury [31].

However, application of any internal load monitoring program should expand beyond just physical training and encompass other forms of incidental loading (e.g., drill, field exercises) in order to address any training program induced cumulative load [32]. Due to the fact that the recruit training course has a specific end-point which recruits must meet with regard to assessed levels of fitness and strength, chronic loading programs prior to recruitment may be useful in preventing injuries to ensure the sudden increase in training load at the commencement of initial training does not lead to too great an increase in training load. This approach has been used by the Royal Military College of Australia, where, following the candidates acceptance at the officer selection board, they were provided with a bridging program to develop and maintain fitness prior to their commencement of training [33]. The results of this program led to an increase in fitness over the bridging period and a reduction in failure rates during testing on arrival.

Since injury patterns are similar between full-time and part-time army recruits during basic training, injury prevention approaches that benefit one population should benefit the other. Following evidence-based practice in this area, physical training instructors should monitor movement quality and lower limb kinematics using validated tools in army recruits to identify where corrective training may be useful. Efforts should also be made to provide opportunities for physical training instructors to increase their experience and qualifications. Basic training programs need to have training load incrementally increased and recruits should therefore be required to have a documented chronic training history prior to the start of basic training, to minimise injury risks. ‘At risk’ recruits should be screened further for risk of injury, if they perform poorly on fitness assessments.

There are some limitations to this study. Given the retrospective, self-reporting nature of this reporting system, there is potential for recall bias, or as reported previously [34], for injuries to go completely unreported. Recommendations have therefore been made for a hybrid system which utilises both point of care and individual reporting features to alleviate this issue [5]. In addition, as the individual is responsible for classification of the details of an injury (i.e. location, nature, mechanism, etc), there may have been some misclassification of injuries, without the researcher’s knowledge. In addition, the relatively small numbers of injuries, especially in ARES personnel, meant there were wide 95% confidence intervals in estimates associated with some categories.

## Conclusion

Types and causes of injuries reported by army recruits as having occurred during basic training are similar between full-time and part-time elements of the army. Both service types are injured at a similar rate, and at a higher rate than in the rest of their careers. Both full-time and part-time personnel undertaking basic training are most commonly injured during physical training. Their injuries tend to be soft-tissue in nature and the knee is the body site most affected by injuries in this context, with a large proportion of injuries stemming from overuse. Proposed interventions for both full-time and part-time army recruits should consider these findings and key points raised in the preceding discussion.

## Abbreviations

ARA: Australian Regular Army
ARES: Army Reserve
FMS: Functional Movement Screen
FT: Full Time
IR: Injury Rates
IRR: Injury Rate Ratio
MPI: Minor Personal Injury
PT: Part Time
RPE: Rating of Perceived Exertion
SPI: Serious Personal Injury
TOOCS: Type Of Occurrence Classification System
WHSCAR: Workplace Health, Safety, Compensation and Reporting
